# In‐solution antibody harvesting with a plant‐produced hydrophobin–Protein A fusion

**DOI:** 10.1111/pbi.12780

**Published:** 2017-08-01

**Authors:** Katri Kurppa, Lauri J. Reuter, Anneli Ritala, Markus B. Linder, Jussi J. Joensuu

**Affiliations:** ^1^ VTT Technical Research Centre of Finland Ltd. Espoo Finland; ^2^ Aalto University Department of Biotechnology and Chemical Technology Espoo Finland

**Keywords:** antibody, hydrophobin, *Nicotiana benthamiana*, Protein A, purification, tobacco BY‐2 suspension cells

## Abstract

Purification is a bottleneck and a major cost factor in the production of antibodies. We set out to engineer a bifunctional fusion protein from two building blocks, Protein A and a hydrophobin, aiming at low‐cost and scalable antibody capturing in solutions. Immunoglobulin‐binding Protein A is widely used in affinity‐based purification. The hydrophobin fusion tag, on the other hand, has been shown to enable purification by two‐phase separation. Protein A was fused to two different hydrophobin tags, HFBI or II, and expressed transiently in *Nicotiana benthamiana*. The hydrophobins enhanced accumulation up to 35‐fold, yielding up to 25% of total soluble protein. Both fused and nonfused Protein A accumulated in protein bodies. Hence, the increased yield could not be attributed to HFB‐induced protein body formation. We also demonstrated production of HFBI–Protein A fusion protein in tobacco BY‐2 suspension cells in 30 l scale, with a yield of 35 mg/l. Efficient partitioning to the surfactant phase confirmed that the fusion proteins retained the amphipathic properties of the hydrophobin block. The reversible antibody‐binding capacity of the Protein A block was similar to the nonfused Protein A. The best‐performing fusion protein was tested in capturing antibodies from hybridoma culture supernatant with two‐phase separation. The fusion protein was able to carry target antibodies to the surfactant phase and subsequently release them back to the aqueous phase after a change in pH. This report demonstrates the potential of hydrophobin fusion proteins for novel applications, such as harvesting antibodies in solutions.

## Introduction

Antibodies are essential in modern medicine as diagnostic agents and in targeted drug delivery. Being the fastest growing area of the pharmaceutical industry, monoclonal antibodies (mAbs) are estimated to reach a total market size of 125 billion US$ by 2020 (Ecker *et al*., 2015). MAbs are mainly produced in animal cell cultures, where they are secreted to the culture media. The industrial standard for harvesting mAbs involves an initial Protein A‐based affinity chromatography step. Despite their widespread use, chromatographic methods suffer from difficulties in scalability. The system relies on batch operation, and transfer to continuous mode is not possible. It is a multistep, labour‐intensive process that represents a major part of the overall production costs. Alternative procedures include two‐phase extraction using conventional salt–polymer systems, for example polyethylene glycol (Azevedo *et al*., 2009). The drawback of these rather simple systems is often poor reproducibility due to sensitivity to, for example, temperature, contaminants or salt concentration (Collén *et al*., 2002).

Here, we describe a bifunctional fusion protein, produced in plants, which may enable a novel, low‐cost and easily scalable strategy for antibody harvesting in solutions. Our approach is inspired by two proteins with specific properties: *Trichoderma reesei* hydrophobins (HFBs) and *Staphylococcus aureus* Protein A.

HFBs are small globular proteins which display extreme surface activity due to their unique amphipathic structure (Linder, 2009; Wessels, 1994; Wosten and Scholtmeijer, 2015). They are found in filamentous fungi, where they fulfil a range of biological functions. Secreted HFBs facilitate penetration of water–air interfaces by decreasing surface tension and coat the hypha and spores decreasing wettability, improving dispersion and providing surface adhesion. The versatile biological roles of HFBs have generated a multitude of potential uses in biotechnology, from structure‐enhancing food additives to coating of sensors, nanoparticles and medical instruments (Wosten and Scholtmeijer, 2015).

HFBs are grouped into two classes according to their hydropathy plots. In this work, we focus on the class II hydrophobins HFBI and HFBII. HFBs show a distinct structure comprising a hydrophobic patch at one end of the molecule and a hydrophilic surface at the other (Hakanpää *et al*.,2006a,b). Due to this unique structure, the hydrophobins self‐assemble at liquid–liquid, liquid–solid or liquid–air interfaces to form monolayers (Linder, 2009; Linder *et al*., 2002; Szilvay *et al*.,^2007^). Their amphipathic nature also allows hydrophobins to interact with small molecule surfactants. This property is commonly used in the purification of hydrophobins and hydrophobin fusion proteins by aqueous two‐phase separation (ATPS) (Collén *et al*., 2002; Joensuu *et al*., 2010; Linder *et al*., 2001).

Protein A is an antibody‐binding protein widely used in affinity chromatography. It reversibly binds antibodies of the IgG class (IgG1, IgG2, IgG4, IgG3). Based on the number of binding sites, a Protein A molecule can bind up to five IgG molecules (Uhlen *et al*., 1984). However, experimental data suggest that the ratio of Protein A to IgG is closer to 1 : 2 (Yang *et al*., 2003). In most applications, the Protein A is chemically bound to a solid chromatography matrix. The antibodies are released from Protein A by decreasing the pH.

We set out to engineer a fusion protein combining two active blocks, HFB and the immunoglobulin‐binding domain of Protein A, in the same polypeptide chain. We expected the novel bifunctional protein to bind mAbs effectively in solution, but also to be separated in a water‐surfactant two‐phase extraction system. Hence, the fusion protein may be used to capture antibodies from solution and concentrate them to the surfactant phase. The phase separation can be performed in a single vessel, by addition of the antibody‐capturing fusion protein and a surfactant. The whole process requires only liquid handling and is therefore easily scalable without complex equipment. A similar two‐phase system utilizing the Protein A–IgG interaction was recently reported by McLean *et al*. (2012). Whereas their two‐phase system was formed intrinsically by an oleosin‐tag fused to the Protein A moiety, we chose a strategy utilizing external two‐phase system based on nonionic surfactant to allow case‐sensitive optimization of purification conditions in a more flexible manner.

HFB fusion proteins have been produced in filamentous fungi (Linder *et al*., 2004; Mustalahti *et al*., 2013), insect cell cultures (Lahtinen *et al*., 2008), plants (Gutiérrez *et al*., 2013; Jacquet *et al*., 2014; Joensuu *et al*., 2010; Pereira *et al*., 2014; Phan *et al*., 2014; Saberianfar *et al*., 2015) and in plant cell cultures (Reuter *et al*., 2014). Whereas production of HFB fusion proteins has been challenging in some other hosts, plants have shown to be an especially suitable production platform. The HFB fusion strategy has, in some cases, significantly enhanced accumulation of the recombinant proteins (Jacquet *et al*., 2014; Joensuu *et al*., 2010). This effect has been attributed to HFB‐induced formation of protein bodies in the host cells (Conley *et al*., 2011; Joensuu *et al*., 2010). In plants, the fusion proteins are not only accumulated in high yields, but are also correctly folded. In addition, plants contain very few native proteins that would be co‐purified in ATPS lowering the product purity (Joensuu *et al*., 2010; Reuter *et al*., 2014). Furthermore, field grown transgenic plants may provide an ideal low‐cost production platform for commodity proteins aimed at biotechnological applications outside the pharma industry (Fischer *et al*., 2013). However, contained production might be necessary for some applications, and regulatory issues may apply. Both transient expression systems and plant cell cultures may be contained and provided adherence to cGMP requirements (Fischer *et al*., 2012; Ritala *et al*., 2014). Considering the downstream processing, suspension cell cultures may provide better overall cost efficiency.

Our goal in this study was to demonstrate a proof of principle for in‐solution antibody harvesting using a novel bifunctional fusion protein. We also evaluated production of the fusion proteins both in *Nicotiana benthamiana* plants and in tobacco BY‐2 suspension cells.

## Results

### Screening for a hydrophobin fusion strategy

**Figure 1:**
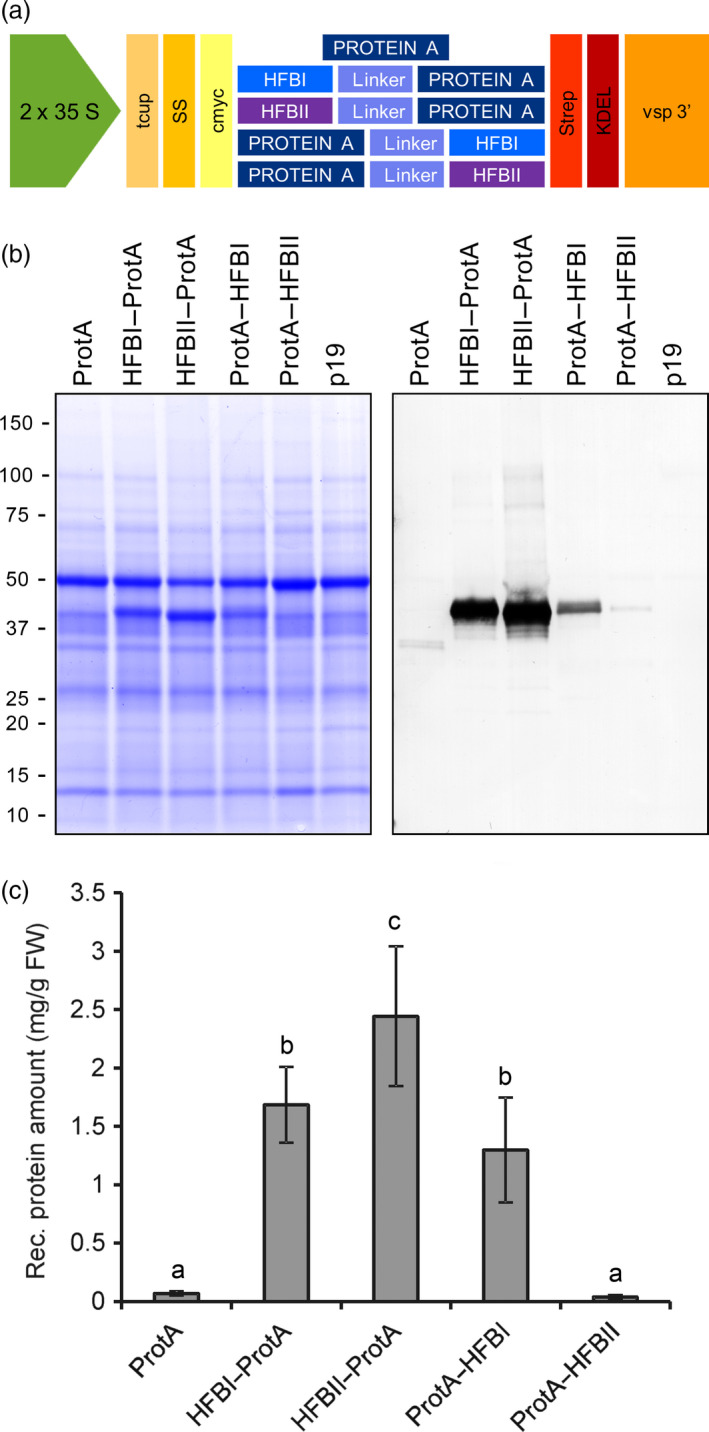
Transient expression of Protein A and HFB fusions in *N. benthamiana*. (a) Schematic presentation of gene constructs of Protein A and fusions with HFBI or HFBII. (b) Pooled samples analysed on Coomassie‐stained SDS–PAGE and (c) on Western blot. (d) Recombinant protein yields analysed as band intensities from Western blots. Error bars indicate standard error of mean (n = 6). The letters indicate significant difference (*P *<* *0.05).

We used agro‐infiltrated *Nicotiana benthamiana* plants to screen for the best hydrophobin fusion strategy. Protein A was constructed in the same polypeptide chain with HFBI or HFBII in both N‐ and C‐terminal orientations (Figure 1a and Figure S1). The yield of N‐ and C‐terminal HFBI fusions reached 1.7 ± 0.3 and 1.3 ± 0.5 mg/g of fresh leaf material (mean±SE, n = 6) (Figure 1b). The HFBII–Protein A accumulated better than the HFBI fusions, 2.4 ± 0.6 mg/g fresh leaf material or 24.3 ± 6.9% of TSP. This represented an approximately 35‐fold increase in yield in comparison with nonfused Protein A. However, the yield of Protein A‐HFBII remained on a similar level to that of the nonfused Protein A. Due to consistent expression levels, we used only the N‐terminal fusions, HFBI–Protein A and HFBII–Protein A, in further experiments.

### Subcellular localization

**Figure 2:**
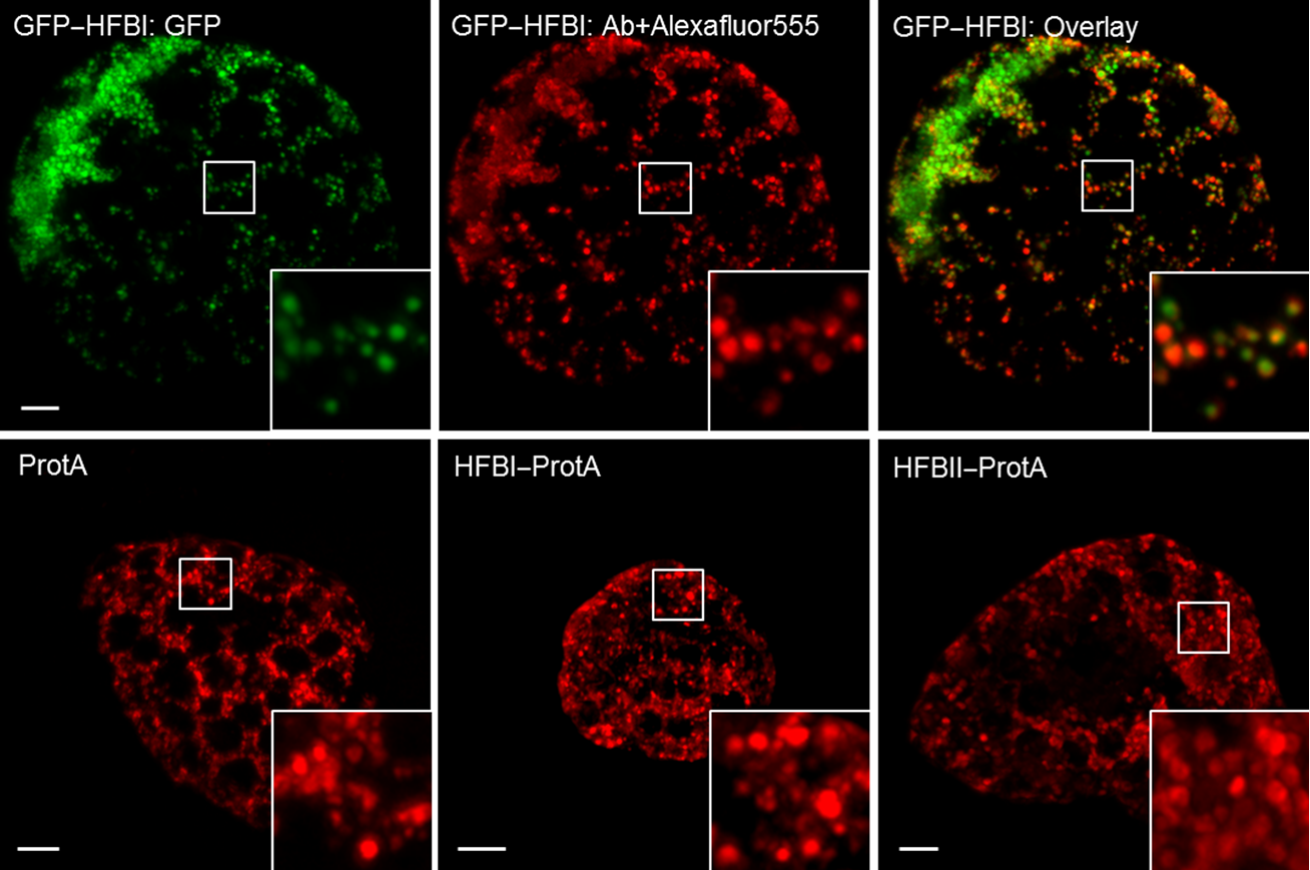
Immunostained confocal microscopy images of *N. benthamiana* protoplasts showing subcellular localization of recombinant proteins. Upper panel: GFP–HFBI was used as a positive control. On the left, GFP‐derived signal shows a typical morphology of HFBI‐induced protein bodies. In the middle, the same cell immunostained with anti‐c‐Myc primary antibody and Alexafluor^®^555‐conjugated secondary antibody. On the right, an overlay image. No signal was detected from the same sample treated without the primary antibody. Lower panel: representative images of protoplasts expressing Protein A (left), HFBI–Protein A (middle) and HFBII–Protein A (right). Protein body‐like structures, similar in size and shape, can be seen in all samples. All images are maximum intensity projections of z‐stack images. Scale bars indicate 5 μm.

We studied the subcellular localization of the ER‐targeted recombinant proteins by immunofluorescent microscopy of protoplasts prepared from agro‐infiltrated leaves (Figure 2). GFP–HFBI fusion protein, known to accumulate in protein bodies (Joensuu *et al*., 2010), served as a positive control. The GFP–HFBI‐induced protein bodies were visible both in intact leafs (not shown) and in the fixed protoplasts (Figure 2). The protein bodies were visualized equally well by the GFP as by the signal derived from the fluorescent probe binding to c‐Myc tag. Protein A, both fused and nonfused, focused similarly into protein body‐like structures. We observed no apparent difference between the constructs. However, the bodies were less abundant and slightly more scattered than the GFP–HFBI‐induced protein bodies.

### Aqueous two‐phase separation

**Figure 3:**
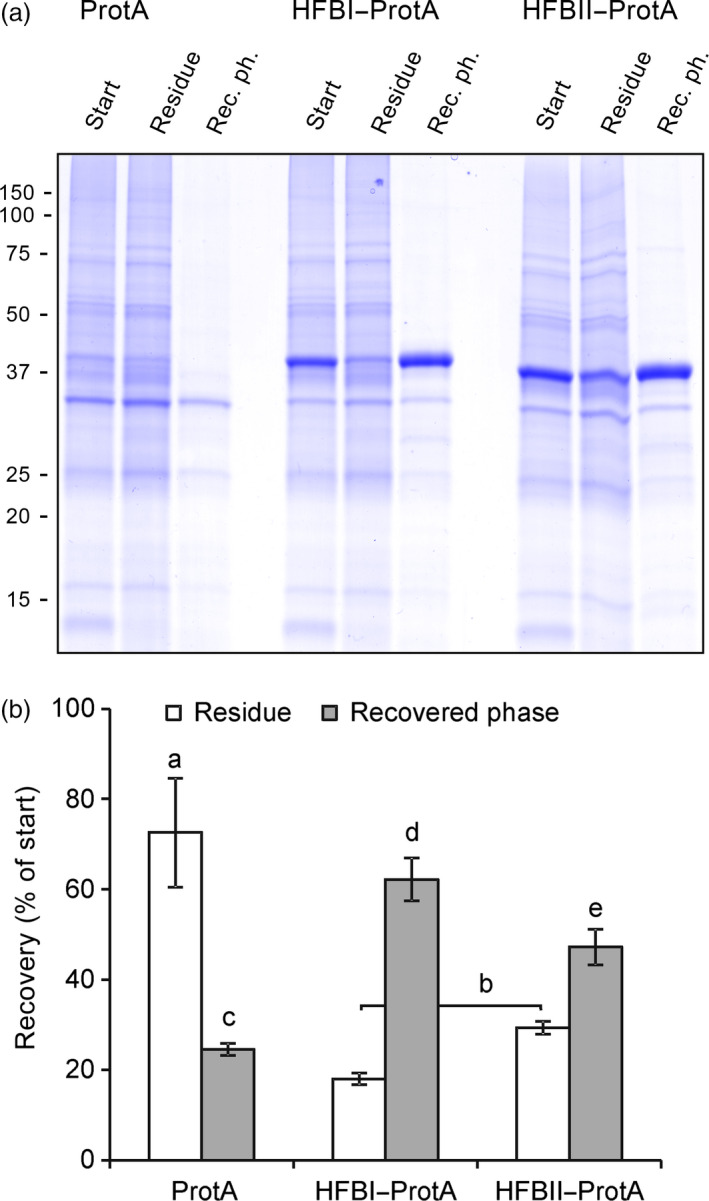
Fusion proteins retain the amphipathic properties of the HFB block. (a) A Coomassie‐stained SDS–PAGE of pooled samples from three replicates shows that both fusion proteins partitioned to the surfactant and were found in recovered phase, whereas the nonfused Protein A remained mainly in the residue as did most native plant proteins. Equal volumes of samples were loaded on gel. Fraction volumes are presented in Figure S2. (b) Recovery rate of the proteins in residue and in the recovered phase analysed on a Western blot. Letters indicate significant difference (n* *=* *3, *P *<* *0.05). Error bars indicate standard deviation.

Next, we examined the amphipathic properties of the HFB blocks by performing ATPS using two fusion constructs, HFBI–Protein A and HFBII–Protein A (Figure 3). The partition coefficient (*k*) describes the ratio of the protein concentration between surfactant phase and residue. Both HFBI–Protein A and HFBII–Protein A displayed regular hydrophobin‐like partitioning in the two‐phase system resulting in *k*‐values of 4.8 ± 0.9 and 2.4 ± 0.6, respectively (mean±SD, n = 3), whereas the nonfused Protein A did not partition into the surfactant (*k *=* *0.4 ± 0.1). The overall recovery rate of HFBI–Protein A (62 ± 5%) was significantly better than that of HFBII–Protein A (47 ± 4%) or nonfused Protein A (25 ± 1%).

### Antibody‐binding capacity of the hydrophobin–Protein A fusion proteins

**Figure 4:**
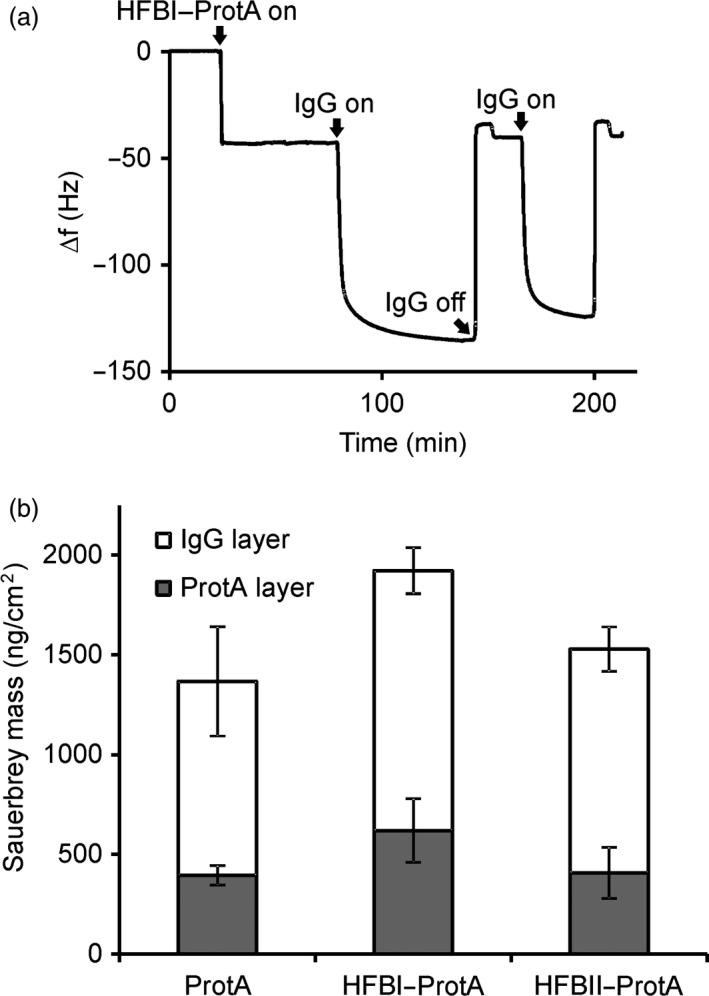
Fusion proteins retain the reversible antibody‐binding capacity of the Protein A block. (a) The QCM‐D experiment showed reversible antibody binding to the HFBI–Protein A layer, represented as a function of time and oscillation frequency. Protein binding reduced the oscillation frequency of the polystyrene‐coated quartz crystal. The curve shows binding of HFBI–Protein A (20 min time point) and of IgG (80 min) and release of IgG by decreasing buffer pH to 2.2 (140 min). The procedure was repeated twice. (b) A similar experiment shows that surface‐bound Protein A, HFBI–Protein A and HFBII–Protein A (grey bars) all bind rituximab with similar capacities (white bars). The error bars indicate standard deviation between repeated measurements.

Having confirmed that the fusion proteins could be separated in ATPS, we set out to study the antibody‐binding capacity of the Protein A block. Antibody binding was measured using a quartz crystal microbalance with dissipation monitoring (QCM‐D). The QCM‐D measures the change in oscillation frequency as a substance is bound to the surface of a quartz crystal. The frequency change is related to the mass of the bound thin layer via the Sauerbrey equation (Höök *et al*., 2001). The surface‐bound layer dampens the oscillation frequency of the freely oscillating crystal. This effect is described by the dissipation factor and depicts the structure of the bound layer. Commercially available Protein A served as a reference for HFBI–Protein A and HFBII–Protein A. All three proteins formed reproducible and stable thin layers on the polystyrene surface (Figure 4b, bottom bars). To evaluate the IgG binding capacity of the fusion proteins, a solution of the rituximab antibody was applied to the protein layers. Addition of the antibody resulted in a mass increase that was similar in the case of all three proteins (Figure 4b, top bars). The molar ratios of rituximab bound to the immobilized fusion proteins were estimated on the basis of the Sauerbrey masses obtained from the QCM‐D data. One mole of immobilized HFBI–Protein A bound 1.5 ± 0.3 (mean ± SD, n = 3) moles of rituximab. The corresponding figure for HFBII–Protein A was slightly lower, 1.2 ± 0.5. The molar ratio of the commercial Protein A to rituximab was 1.2 ± 0.3. No specific antibody binding was observed on layers of nonfused HFBI (data not shown) or BSA (Figure S2). The results confirmed that both fusion proteins retained the immunoglobulin‐binding capacity of the Protein A block.

To demonstrate the release of antibodies and regeneration of the antibody‐binding layer, we performed two successive rounds of IgG binding and release using commercial IgG λ antibodies. Release of the bound IgG λ was accomplished by decreasing the pH by rinsing the layer with acidic buffer (Figure 4a). When glycine buffer at pH 2.2 was introduced to the surface‐bound HFB–Protein A/IgG λ complex, the mass decreased instantly. The released mass corresponded to the amount of antibody initially bound. After elevating the pH to 8, the layer was capable of rebinding the IgG λ without a significant decrease with respect to the initial amount. The HFB–Protein A layers remained stable and capable of binding IgG λ after overnight incubation in buffer (data not shown).

### Antibody capture from hybridoma culture supernatant

**Figure 5:**
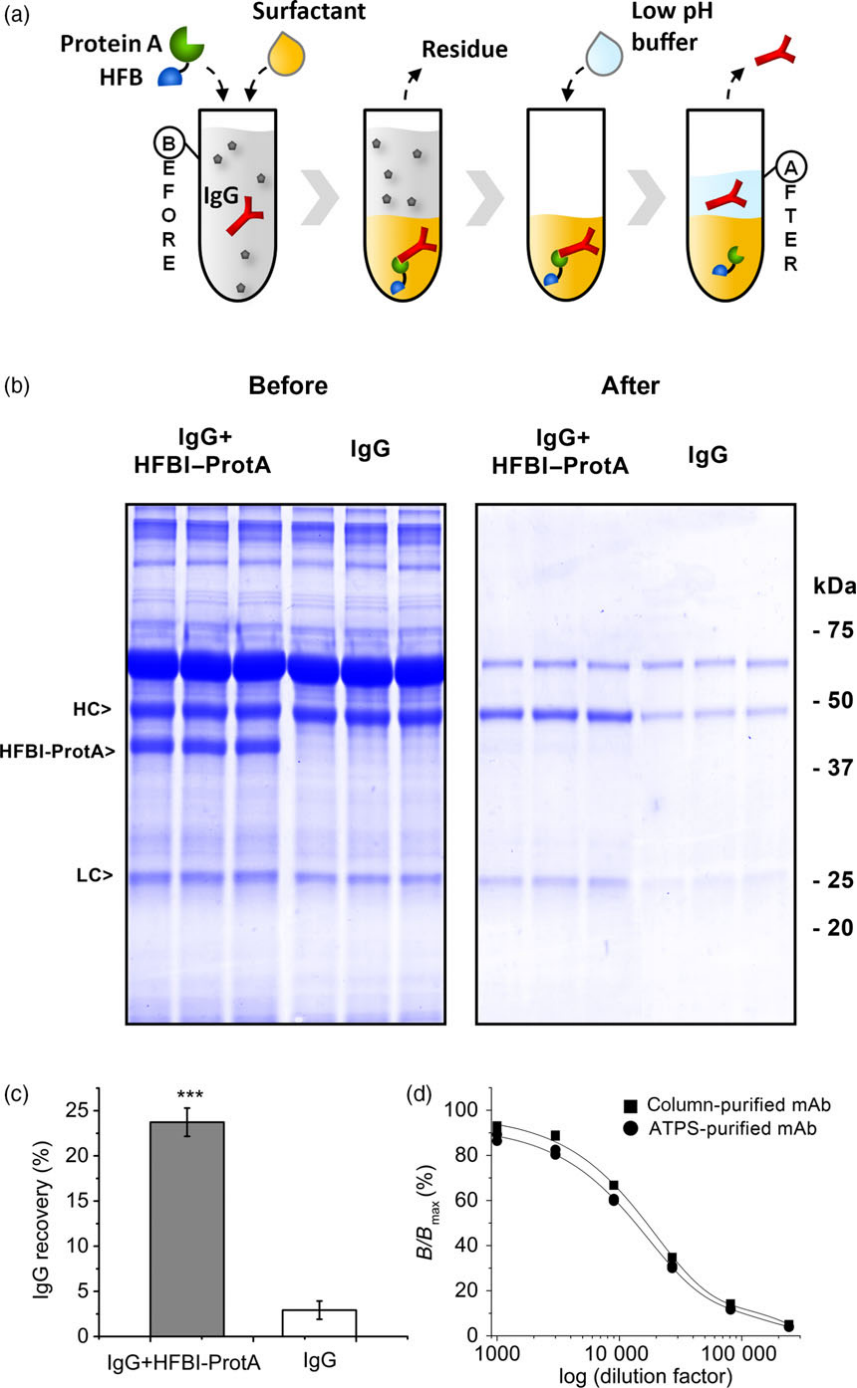
The HFBI–Protein A fusion protein can capture antibodies from hybridoma culture supernatant. (a) The concept of the in‐solution antibody harvesting. The Protein A block (green) binds to the IgG (red) when added to the antibody‐containing culture supernatant (before). Addition of a surfactant (tan) results in a two‐phase system. The HFB block (blue) guides the HFBI–Protein A/IgG complex to the surfactant phase. The aqueous residue is discarded. The IgG is released by addition of acidic buffer and recovered from the aqueous phase (after). (b) SDS–PAGE showing the partition of the IgG from hybridoma culture supernatant in ATPS process with the HFBI–Protein A (left) and without (right). (c) Overall recovery of IgG from hybridoma culture supernatant. The error bars represent standard deviation of the mean (n* = *3). The asterisks indicate significant difference (*P *>* *0.001). (d) Bioactivity of ATPS‐purified IgG (round) compared to IgG purified with Protein A column chromatography (square). The *x*‐axis represents the logarithm of the antibody sample dilution factors.

After confirming the bifunctionality of the fusion proteins, that is, the IgG binding capacity of the Protein A block and the amphipathic properties of the HFB block, we proceeded to demonstrate the principle of antibody capture in ATPS process (Figure 5a). Here, we used only HFBI–Protein A, as it outperformed HFBII–Protein A in the initial ATPS and IgG binding experiments. Hybridoma culture supernatant containing a monoclonal anti‐Chlamydia IgG antibody was mixed with the nonionic detergent Triton X‐114 and HFBI–Protein A. After establishing the two‐phase system, the residual aqueous phase was removed. The antibodies were released from HFBI–Protein A by addition of acidic buffer to the detergent phase, and after second‐phase separation, the antibodies were collected from the aqueous phase. With this solution based capture, 24 ± 2% (mean±SD, n = 3) of the antibody was recovered from the hybridoma culture supernatant while the antibody recovery rate in the absence of the fusion protein was significantly lower, 3 ± 1% (Figure 5c). The purification procedure cleared most of the host cell proteins present in the culture supernatant (Figure 5b). The bioactivity of the ATPS‐purified antibody was studied by a fluoroimmunoassay (Figure 5d). The same mAb purified with traditional Protein A column chromatography was used as a reference. The antigen binding capacity of the ATPS‐purified antibody was 81%–103% (n = 2) compared to the chromatographically purified one, the result being within the approval limits of a commercial product.

### Contained protein production in BY‐2 suspension cells

**Figure 6:**
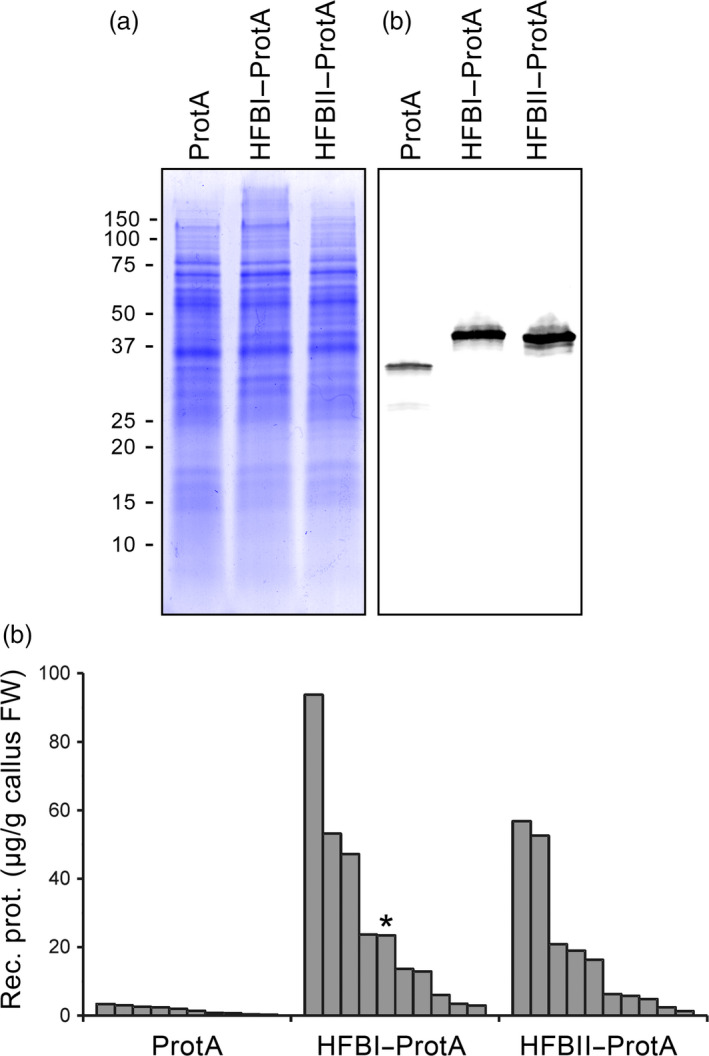
Accumulation of Protein A, HFBI–Protein A and HFBII–Protein A in tobacco BY‐2 cell cultures. (a) A Coomassie‐stained SDS–PAGE and (b) a Western blot illustrating the accumulation of the recombinant proteins in samples pooled from 10 callus clones for each construct. The Western blot is visualized using anti‐c‐Myc antibodies. (c) Amount of recombinant proteins in the 10 best callus clones for each construct determined from Western blots. The line used to initiate a suspension culture is indicated with an asterisk.

Having established the good expression levels in *N. benthamiana* and demonstrated the functionality of the HFBI–Protein A, we evaluated the possibility to produce the fusion proteins in transgenic BY‐2 cells. After preliminary screening of callus lines, protein accumulation was quantified for the 10 best clones expressing Protein A, HFBI–Protein A and HFBII–Protein A (Figure 6). Nonfused Protein A yielded on average approximately 2 μg/g of fresh callus, whereas both HFBI and HFBII fusions boosted the average accumulation approximately 10‐fold to 20 to 30 μg/g fresh callus (Figures 6a,b). It should be noted, however, that the accumulation levels between the best 10 clones of each line showed considerable variation (Figure 6c). This is most probably due to random insertion sites in the genome and effect of the location to the transcriptional activity.

**Figure 7:**
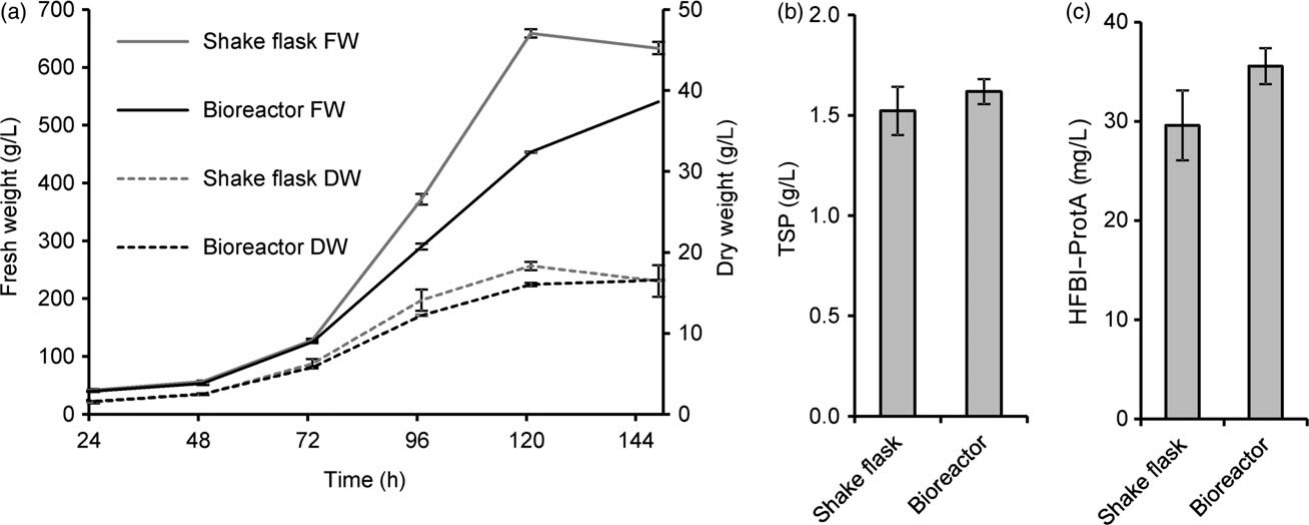
HFBI–Protein A producing BY‐2 suspension cell culture propagated in 30‐litre culture volume. (a) Accumulation of dry mass was similar in shake flasks and in the bioreactor. The error bars represent standard deviation between three biological replicates in shake flasks and three technical replicates in the bioreactor. (b) The accumulation of total soluble protein, analysed by Bradford assay, and (c) the recombinant protein, analysed from Western blots, were comparable in the bioreactor and shake flask cultivations. The error bars represent standard deviation between three technical replicates.

Based on favourable growth characteristics and homogeneity of the callus, we selected a clone expressing HFBI–Protein A to be grown in suspension culture in shake flasks and subsequently in a stirred tank bioreactor in 30 l scale. The accumulation of biomass (dry weight) in the bioreactor was comparable to that in shake flasks (Figure 7a). The yield of HFBI–Protein A reached 30 ± 6 mg/l (mean±SD, n = 3) and 36 ± 3 mg/l in shake flasks and bioreactor, respectively (Figure 7c). To establish a streamlined downstream process suitable for large‐scale production, the whole culture suspension was homogenized in a high‐pressure homogenizer and clarified by centrifugation. The clarified extract was directly applied to ATPS, resulting in partially purified protein extract with HFBI–Protein A concentrated to 44 ± 2 mg/l with recovery rate of 49 ± 10% (mean±SD, n = 3). Thus, the total yield after first purification was approximately 18 mg HFBI–Protein per litre of culture volume.

## Discussion

Monoclonal antibodies have a key role in modern medicine, research and diagnostics. In many cases, however, the high costs of production are limiting their use. The production cost becomes an issue especially now as the first generic antibody drugs are entering the market. Harvesting and initial purification of antibodies using chromatographic methods poses a major bottleneck and represents a large part of the overall production cost (Farid, 2007; Raven *et al*., 2015). The aim of this study was to show that the use of a HFB tag can be broadened to include not only purification of fusion proteins themselves, but also of noncovalently bound target molecules, such as antibodies. We constructed a bifunctional fusion protein from two blocks: Protein A and either HFBI or HFBII. The fusion proteins were produced in *Nicotiana benthamiana* plants and in BY‐2 suspension cells. The best‐performing fusion protein was finally tested for capturing monoclonal antibodies from hybridoma culture supernatant.

HFB‐fused Protein A reached excellent production yields in *N. benthamiana*. Both N‐terminal HFB fusion tags improved accumulation in comparison with nonfused Protein A up to 35‐fold. We observed the same trend later in BY‐2 calli, although the accumulation levels varied between the clones. HFBI fused to either the N‐ or C‐terminus of the Protein A improved the accumulation to similar levels in *N. benthamiana*. HFBII, however, enhanced the accumulation of Protein A only as an N‐terminal fusion, whereas the C‐terminal fusion accumulated to levels similar to those observed with nonfused Protein A. The N‐terminus of the HFBII, before the first disulphide bridge, is four amino acids shorter than that of HFBI (Sunde *et al*., 2008). This may cause a steric hindrance for correct folding of the Protein A‐HFBII and thus limit its accumulation. The HFBI fusion has previously been reported to enhance the accumulation of some fusion proteins in plants (Gutiérrez *et al*., 2013; Jacquet *et al*., 2014; Joensuu *et al*., 2010). However, this effect has not been consistent and several studies have shown no improvement in yields (Pereira *et al*., 2014; Phan *et al*., 2014). This is the first report on improved product accumulation in BY‐2 cells using a HFB tag.

The yield‐enhancing effect of HFB fusion tags has been attributed to the formation of protein bodies (Conley *et al*., 2011; Joensuu *et al*., 2010). We examined the subcellular localization of the fusion proteins by immunofluorescent confocal microscopy of protoplasts prepared from agro‐infiltrated *N. benthamiana* leaves. Interestingly, we found that all Protein A constructs accumulated in protein body‐like structures, regardless of the HFB fusion. When compared to GFP–HFBI‐induced protein bodies, the Protein A induced bodies appeared to be less abundant, but were similar in size. We observed no apparent differences in localization of fused or nonfused Protein A. Previous reports have suggested that protein bodies would form independently of the presence of HFBs when the recombinant proteins accumulate in levels higher than 0.2% of TSP (Gutiérrez *et al*., 2013; Saberianfar *et al*., 2015). Here, the yields of all recombinant proteins exceeded that threshold. Thus, these results support the conclusions of the previous studies that formation of protein body‐like structures may indeed be largely a concentration‐dependent phenomenon. However, in our experiment, even the 35‐fold difference in accumulation levels of fused and nonfused Protein A did not result in apparent differences in number or size of the protein bodies. Therefore, the formation of protein bodies alone may not be the only reason for increased accumulation. This challenges the previous assumption and leaves open the question of other possible yield‐increasing mechanisms of the HFB fusion. However, this question was outside the scope of this study.

In the future, transgenic plants grown in the field may provide an ideal low‐cost production platform for HFBI–Protein A and other commodity proteins. However, contained production might be a necessity for some applications, especially in the case of pharmaceutical targets (Fischer *et al*., 2012; Ritala *et al*., 2014). In comparison with *N. benthamiana‐*based transient production systems, plant suspension cells may prove to be a useful alternative. As demonstrated here and in previous studies, BY‐2 cell lines can be propagated in conventional industrial‐scale bioreactors and the downstream processing is readily scalable (Raven *et al*., 2015; Reuter *et al*., 2014). Low productivity is nevertheless an issue. Yields in plant cell cultures typically vary from 0.005 to 200 mg/l, and a yield in range of 10 mg/l is generally considered satisfactory for starting commercial product development (Hellwig *et al*., 2004). Thus, the productivity of the suspension culture here was on a good level (36 mg/l). Nevertheless, an approximate calculation indicates that the 30 litre culture volume correlated in yield to only ca. 40 *N. benthamiana* plants. However, it should be noted that the yield of HFBI–Protein A in transient expression was very high, whereas the potential to increase productivity of the BY‐2 suspension culture remains vast. We have previously reported 10‐fold increase in productivity with a stable model protein GFP–HFBI in BY‐2 suspension cells (Reuter *et al*., 2014). Several means for improving the productivity of BY‐2 suspension cells have been published recently, including improved culture media (Holland *et al*., 2010), FACS‐based clone screening (Kirchhoff *et al*., 2012), protease knockout lines (Mandal *et al*., 2014) and development of culture systems (Raven *et al*., 2015). However, improving the yield in the BY‐2 suspension cells was not the aim of this study.

We expected the fusion proteins to exhibit two functions. First, they should demonstrate the amphipathic properties of hydrophobins and be efficiently separated into a surfactant phase from aqueous solution. Second, they should reversibly bind immunoglobulins. The initial ATPS experiment showed that both HFBI–Protein A and HFBII–Protein A partitioned well to surfactant phase. The HFBI–Protein A, however, partitioned slightly better than HFBII–Protein A (Figure 3). To examine the antibody‐binding capacity of the fusion proteins, we used a quartz crystal microbalance with dissipation monitoring (QCM‐D). Both fusion proteins bound IgG with similar efficiency to that of commercial Protein A. According to the literature, the wild‐type Protein A could theoretically bind to five immunoglobulins (Uhlen *et al*., 1984), but the experimental data, as well as information from chemical providers, suggest that the real rate is more close to 1 : 2 ratio. Although this potential rate was not reached in this experiment, we conclude that the HFB block does not hinder the antibody‐binding capacity of the fusion proteins. The fusion proteins also retained the capability of Protein A to repeated rounds of antibody binding and release by adjusting the pH. Thus, the fusion protein could be potentially reused in a recyclable system, thus lowering the purification costs.

Having separately confirmed the two functions of the fusion protein, we put the HFBI–Protein A to a final test to see whether it could be used to harvest antibodies from technologically relevant hybridoma culture supernatant. The ATPS experiments demonstrated that the antibody was bound by the Protein A block and carried to the surfactant phase by the HFB block of the fusion protein. Furthermore, the antibody could be recovered back to the aqueous phase by decreasing the pH.

The recovery rate of the antibody was somewhat lower than would have been expected on the basis of separation of HFB–Protein A alone. The vastly larger size and relatively hydrophilic nature of the HFBI–Protein A/IgG complex in comparison with the smaller and sufficiently amphipathic fusion protein alone may have hindered the separation. It must also be noted that in the case of antibody purification procedure, two consequent phase separations follow each other and may explain the poor Ab recovery. Binding of the fusion protein to the IgG could also have been hindered by multimerization of the fusion protein due to self‐assembly tendency of the HFB block (Linder *et al*., 2002). Regarding the antibody purity, the major carry‐over protein remaining in the recovered antibody fraction was most likely bovine serum albumin (Figure 5b) used as a stabilization agent in the hybridoma fermentation media. A fraction of the antibody (3%) was recovered from the ATPS also without HFBI–Protein A. Some of the antibodies may have migrated to the surfactant phase due nonspecific hydrophobic interactions with the surfactant or passive distribution between the phases. Similarly, the antibody may have migrated back to the acidic buffer. Nevertheless, the difference to recovery rate using HFB–Protein A was sufficient for proof of concept. Furthermore, the antigen binding capacity of the purified antibody was not compromised by the ATPS procedure.

Whereas the experiments yielded merely a qualitative demonstration, further optimization of the process could result in a feasible, recyclable antibody purification system. Options for tuning and optimization of the system are versatile with respect to choice of surfactant, additives and buffer composition. Further work to improve the affinity of the fusion protein and the purification conditions is ongoing.

This report makes a case for novel applications of HFBs beyond their use as a fusion tag simply to aid production and purification of recombinant proteins. The bifunctional fusion protein, inspired by the unique properties of the HFBs, may open novel applications for antibody harvesting and purification. However, the applications are not limited to that. Recently, the surface‐active and self‐assembling properties of HFBs and HFB fusion proteins have been utilized for example in functional coatings of nanoparticles and surfaces (Soikkeli *et al*., 2016; Sarparanta *et al*., 2012; Kurppa *et al*., 2014). With the emerging interest in material technology, HFBs can be seen as very interesting building blocks for a host of novel fusion proteins.

## Experimental Procedures

### Construct design

A codon optimized coding sequence for the immunoglobulin‐binding domain (amino acids 27‐325) of *Staphylococcus aureus* Protein A (accession 1314205A) was synthesized at Genscript (USA). Four potential N‐glycosylation sites were removed (N to Q) (Figure S1). The coding sequence was connected to *HFBI* (accession XM_006964119.1) or *HFBII* (accession P79073) of *Trichoderma reesei* by a (GGGS)_3_ linker as described in Figure 1. The sequence for HFBII was codon optimized. The constructs were assembled and placed in a plant binary expression vector pCaMterX (Harris and Gleddie, 2001) under the control of the dual‐enhancer cauliflower mosaic virus 35S promoter (Kay *et al*., 1987), tcup translational enhancer (Wu *et al*., 2001) and the soya bean (*Glycine max*) vspB (Mason *et al*., 1988) terminator using Golden Gate cloning (Engler *et al*., 2009). The vector incorporates a c‐Myc‐tag and a signal sequence for secretory pathway (Prb1) in the N‐terminus and StrepII tag and ER‐retention signal (KDEL) in the C‐terminus of the open‐reading frame. Refer Figure S1 for complete nucleotide sequences. The expression vectors were transformed into *Agrobacterium tumefaciens* strain EHA105 (Hood *et al*., 1993).

### Transient expression in *Nicotiana benthamiana*, tissue sampling and protein extraction

*A. tumefaciens* cultures were grown in liquid LB‐media overnight. The optical density at 600 nm was adjusted to 0.8 with infiltration buffer (1 mm MES, 1 mm MgSO_4_). The suspension was mixed with (ratio 2 : 1) a suspension of *Agrobacterium* carrying an expression vector for p19 (Silhavy *et al*., 2002). Leaves from six different 5‐ to 6‐week‐old *N. benthamiana* plants were infiltrated using a syringe and sampled six days postinfiltration (dpi) by collecting four leaf discs (Ø 7.1 mm) for each construct.

The leaf discs were stored frozen at −80 °C and homogenized using a Retsch mill (MM301, Haan, Germany). Ice‐cold extraction buffer (phosphate‐buffered saline, PBS; 137 mm NaCl, 2.7 mm KCl, 8.1 mm Na2HPO4, 1.8 mm KH2PO4, 2% sodium ascorbate, 1 mm EDTA, 1 mm PMSF, 1.25 ug/ml leupeptin pH 7.4) was added (300 ul), and the leaf powder was mixed to a slurry. The protein extract was clarified by centrifugation at 16 873 ***g*** for 2 × 5 min at +4 °C; Eppendorf 5418R, (Eppendorf, Hamburg, Germany). The replicates were either analysed separately to obtain data for statistical analysis or pooled together to show representative sample on SDS–PAGE and western blot.

### Protoplast preparation and imaging

Agro‐infiltrated leaves (6 dpi) were cut into thin strips and digested in enzyme solution [1.5% cellulase R10 (Serva Electrophoresis, Heidelberg, Germany), 0.4% macerozyme R10 (Serva Electrophoresis), 0.4 M mannitol, 20 mm KCl, 20 mm MES (pH 5.7), 10 mm CaCl_2_, 5 mm β‐mercaptoethanol] in the dark at RT overnight. Protoplasts were sieved through a 100‐μm mesh and centrifuged for 10 min at 60 ***g*** at 4 °C (Eppendorf 5810R). After washing twice with WI buffer [0.5 M mannitol, 4 mm MES (pH 5.7), 20 mm KCl], the protoplasts were fixed in 4% paraformaldehyde (Sigma‐Aldrich, St. Louis, MO) in WI for 1 h at RT. The membranes were permeated by incubation in 3% IGEPAL CA‐630 (Sigma‐Aldrich) and 10% DMSO (Merck, Billerica, MA) in PBS for 5 min at RT. Nonspecific binding was blocked by incubation in 2% BSA (Sigma‐Aldrich) in PBS for 1 h at RT. Primary antibody against the c‐Myc tag (mouse, A00864, GenScript, Piscataway, NJ) was applied in PBS (1 : 100) and incubated at 4 °C overnight. Secondary antibody, conjugated with Alexafluor^®^555 (goat anti‐mouse, A21422, Life Technologies, Carlsbad, CA), was applied in PBS (1 : 100) and incubated for 2 h at 38 °C. Between each step, the protoplasts were washed 3× with PBS.

Z‐stack images were acquired with a Zeiss LSM 710 laser scanning confocal microscope (Carl Zeiss, Oberkochen, Germany) equipped with a 63× water immersion objective. Excitation with a 488‐nm agron laser was used for GFP, and fluorescence was detected at 495–550 nm. Alexafluor^®^555 was excited with a 543‐nm HeNe laser, and fluorescence was detected at 550–630 nm.

### ATPS and protein purification

Proteins were extracted for purification by homogenizing snap‐frozen agro‐infiltrated leaves in cold extraction buffer (4× buffer volume/leaf weight). The homogenate was clarified by centrifugation (10 min at 3220 ***g*** at 4 °C; Eppendorf 5810R). To precipitate host cell proteins, particularly Rubisco, the supernatant was set up on magnetic stirrer plate and the pH was adjusted to 4.8 by adding HCl. After two minutes, the supernatant was adjusted back to pH 7.2 with NaOH and clarified with a second centrifugation step. For the ATPS, the supernatant was warmed to 24 °C and mixed with Triton X‐114 (6% w/v, Sigma‐Aldrich). After mixing, the phases were allowed to separate in a separation funnel. The lower (detergent‐rich) phase was collected and washed with isobutanol (Sigma‐Aldrich; 10‐fold volume with respect to detergent mass). The aqueous phase was collected, and the buffer was changed to 100 mm Tris–HCl, 150 mm NaCl and 1 mm EDTA (pH 8.0) with 10DG gel filtration columns (Bio‐Rad, Hercules, CA). Finally, the extract was polished by affinity chromatography using a Streptactin macroprep column according to the manufacturers’ protocol (IBA, Göttingen, Germany).

### Transformation and maintenance of BY‐2 cell cultures

Transformation of the BY‐2 cells was performed as described earlier (De Sutter *et al*., 2005). After two passages on selective media, 48 two‐week‐old calli were screened for product accumulation. Ten lines were selected for further experiments. After 3 weeks, the lines were sampled again for quantitative analysis. The lines were further maintained by subculturing at 3‐week intervals on modified MS media (Nagata and Kumagai, 1999) supplemented with 50 ppm kanamycin. Three lines with good expression levels of HFBI–Protein A were grown in suspension cultures of which one was selected for scaling‐up according to product accumulation and growth characteristics. Suspension cultures were maintained in liquid modified MS media supplemented with 50 ppm kanamycin and subcultured weekly.

### Bioreactor cultivation

Bioreactor IF40 (New Brunswick Scientific, Edison, NJ) cultivation was conducted in a total culture volume of 30 l in batch mode by inoculating at 5% (v/v) with a 7‐day‐old suspension from shake flask cultures. The medium, without antibiotics, was prepared and sterilized in the bioreactor. Cultivation was carried out at 28 °C. Dissolved oxygen (DO) was controlled by stirring speed, airflow and vessel overpressure to maintain DO concentration above 20%. As a control, the same line was propagated in 50 ml volume in shake flasks.

The fresh weight was determined by sampling 10.0 ml of culture suspension in a conical tube and weighing the cell pellet after centrifugation for 10 min at 3220 ***g*** (Eppendorf 5810R). The pellet was freeze‐dried to obtain dry weight.

### Protein extraction from BY‐2

Callus samples were stored at −20 °C. For protein extraction, ice‐cold buffer (PBS, 1 mm EDTA) was added 1 : 2 v/w to callus samples thawed on ice and subsequently homogenized using the Retsch mill. For protein extraction from freeze‐dried cell material from suspension cultures, extraction buffer was added to powdered cell material (40 : 1 v/w) and homogenized using the Retsch mill. The protein extracts were clarified by centrifugation for 10 min at 21130 ***g*** at 4 °C (Eppendorf 5424R).

### Protein analysis

Concentration of TSP was measured using the Bradford analysis (1976) with Bio‐Rad reagent (Bio‐Rad). Protein separation was performed by SDS–PAGE on Bio‐Rad Criterion‐TGX and Mini‐PROTEAN precast gels and stained using GelCode^®^ Blue Stain Reagent (Thermo Scientific, Waltham, MA). Protein quantifications were performed either from SDS–PAGE (Figures 3b and 5b) or by western blot analysis (Fig. 1b) after transferring proteins to nitrocellulose membrane using the Trans‐Blot^®^ Turbo™ system (Bio‐Rad). Proteins were visualized with anti‐c‐Myc tag primary antibody (rabbit, A00172, GeneScript) and a secondary antibody for detection (anti‐rabbit‐AP, 170‐6518, Bio‐Rad). For quantification (Figure 1c) and work in BY‐2, a fluorescently labelled secondary antibody (goat anti‐rabbit, IR Dye^®^ 680RD, LI‐COR Biosciences, Lincoln, NB) was used. Detection was carried out with Odyssey CLX densitometer (LI‐COR Biosciences) and Image Studio 2.1 software. Protein quantities were assessed against known concentrations of purified HFBI–Protein A, commercial rituximab (Oriola, Espoo, Finland) or anti‐Chlamydia mAb 6709 (Medix Biochemica, Espoo, Finland).

### QCM‐D

Protein adsorption was measured by QCM‐D (E4 Biolin Scientific). Polystyrene crystals (Biolin Scientific, Göteborg, Sweden) were cleaned according to supplier's protocol. Protein solutions were diluted in buffer M (0.1 M sodium phosphate, pH 7) and pumped for 5 min. Adsorbed surfaces were stabilized 45–60 min and rinsed with buffer M.

Protein samples were diluted as follows: HFB–Protein A 2 μm, IgG1 λ antibodies 0.05 mg/ml, 0.3 μm (Sigma‐Aldrich). In Figure 4a, a 1/3 molar equivalents of wild‐type HFBI was used together with HFBI–Protein A to enhance surface packing. Antibodies were released by rinsing with glycine–HCl buffer (pH 2.2) for 5 min, followed by buffer M (pH 8).

Three replicate binding experiments were conducted (Figure 4b). HFBI–Protein A, HFBII–Protein A and commercial Protein A (Sigma‐Aldrich) were diluted to 0.1 mg/ml (ca. 2 μm). Rituximab IgG was added (82 nm, 0.01 mg/ml) to the adsorbed protein surfaces for 5–7 min. The bound mass was calculated using the Sauerbrey equation Δm =−C·Δf/n_5_ where C = 17.7 ngHz^−1 ^cm^−2^ for a 5 MHz quartz crystal and n_5_ = 5, the overtone number. The values for bound mass were obtained at the buffer rinsing steps by averaging the data over 100 time points (260 s). Dissipation D was used to examine the viscoelastic properties of the bound protein layer. D is defined as *E*_lost_/2π*E*_stored_, where *E*_lost_ is the energy lost during one oscillation cycle and *E*_stored_ is the total energy stored in the oscillator. Molar ratios were calculated using the Saurbrey mass values and molecular weights of 44 kDa (HFBI–Protein A and HFBII–Protein A) and 50 kDa (commercial Protein A).

### Antibody capture by two‐phase extraction

To capture antibodies from the hybridoma culture supernatant, a molar ratio of 2 : 1 between HFBI–Protein A and mAb was used. In detail, 0.22 ml of purified HFBI–Protein A (5.0 mg/ml) was added to 0.4 ml of concentrated hybridoma culture supernatant containing 4.7 mg/ml of anti‐Chlamydia 6709 mAb (Medix Biochemica) and topped to 1 ml with PBS buffer. Prior to the addition of 6% (w/v) Triton X‐114, the mixture was warmed to 24 °C for 5 min. After mixing 15 min at 24 °C in a rotary drum, the phases were separated by centrifugation at 4000 ***g*** for 5 min. The aqueous phase was removed and the surfactant phase was supplemented with equal volume of 0.05 M glycine–HCl, pH 2.2 to release antibodies from HFBI–Protein A (final pH at this point 3.0). The mixture was incubated in the rotary drum at 24 °C for 5 min and centrifuged as above. The aqueous phase containing the purified antibodies was collected and neutralized to pH 8 with 18 μl of 1M Tris–HCl pH 8.5. Fractions of samples before and after the purification procedure were analysed by SDS–PAGE.

### Measuring bioactivity of ATPS‐purified antibodies

To ensure the bioactivity of the purified anti‐Chlamydia antibody 6709 (Medix Biochemica) after ATPS, the mAb was studied with fluorescence immunoassay. Prior to the measurement the protein samples were subjected to buffer exchange to PBS by gel filtration. The antigen binding capacity was compared against a mAb 6709 purified with Protein A column chromatography. Briefly, dilution series were performed from both Protein A and HFBI–Protein A purified mAbs (sample concentrations 1 μg/ml, 333 ng/ml, 111 ng/ml, 37 ng/ml, 12.3 ng/ml and 4.1 ng/ml). To obtain the maximal binding of mAb 6709, the concentration 10 μg/ml of Protein A purified mAb was included in the assay. The dilution series of mAbs (n = 2 at each concentration) were incubated on anti‐mouse IgG microtiter plates (Kaivogen, Turku, Finland) for 1 h. After incubation, the plates were washed three times with washing buffer (PerkinElmer). Europium labelled Chlamydia antigen (Medix Biochemica) was added on each well and incubated for 1 h followed by washings five times with washing buffer. DELFIA Enhancement solution (PerkinElmer) was added to release Europium, which was measured with a time‐resolved fluorometer EnVision Xcite (PerkinElmer) using 340 nm for excitation and 615 nm for emission. The mAb concentration showing 50% of the maximum binding was calculated for both chromatographically purified and ATPS‐purified mAbs. The bioactivity was determined as a ratio between the antibody concentrations at which 50% of the maximum binding was reached (B/B_max_).

### Statistical analysis

Statistical analyses were performed with SPSS Statistic 22.0 (IBM, Armonk, NY) using two‐tailed Student's independent samples t‐test for two samples and one‐way ANOVA test followed by Tukey's HSD for three or more samples, with significance level of 95%.

## Supporting information


**Figure S1** Nucleotide sequences of expression cassettes.

**Figure S2** QCM‐D experiment showing antibody binding to HFBI‐Protein A layer.

## References

[pbi12780-bib-0001] Azevedo, A.M. , Rosa, P.A.J. , Ferreira, I.F. and Aires‐Barros, M.R. (2009) Chromatography‐free recovery of biopharmaceuticals through aqueous two‐phase processing. Trends Biotechnol. 27, 240–247. 19251328 10.1016/j.tibtech.2009.01.004

[pbi12780-bib-0002] Bradford, M.M. (1976) A rapid and sensitive method for the quantitation of microgram quantities of protein utilizing the principle of protein‐dye binding. Anal. Biochem. 72, 248–254. 942051 10.1016/0003-2697(76)90527-3

[pbi12780-bib-0003] Collén, A. , Persson, J. , Linder, M. , Nakari‐Setälä, T. , Penttilä, M. , Tjerneld, F. and Sivars, U. (2002) A novel two‐step extraction method with detergent/polymer systems for primary recovery of the fusion protein endoglucanase I–hydrophobin I. Biochem. Biophys. Acta. 1569, 139–150. 11853968 10.1016/s0304-4165(01)00244-6

[pbi12780-bib-0004] Conley, A.J. , Joensuu, J.J. , Richman, A. and Menassa, R. (2011) Protein body‐inducing fusions for high‐level production and purification of recombinant proteins in plants. Plant Biotechnol. J. 9, 419–433. 21338467 10.1111/j.1467-7652.2011.00596.x

[pbi12780-bib-0005] De Sutter, V. , Vanderhaeghen, R. , Tilleman, S. , Lammertyn, F. , Vanhoutte, I. , Karimi, M. , Inzé, D. *et al*. (2005) Exploration of jasmonate signalling via automated and standardized transient expression assays in tobacco cells. Plant J. 44, 1065–1076. 16359398 10.1111/j.1365-313X.2005.02586.x

[pbi12780-bib-0006] Ecker, D.M. , Jones, S.D. and Levine, H.L. (2015) The therapeutic monoclonal antibody market. mAbs. 7, 9–14. 25529996 10.4161/19420862.2015.989042 PMC4622599

[pbi12780-bib-0007] Engler, C. , Gruetzner, R. , Kandzia, R. and Marillonnet, S. (2009) Golden gate shuffling: A one‐pot DNA shuffling method based on type ils restriction enzymes. PLoS ONE 4, e5553. 19436741 10.1371/journal.pone.0005553 PMC2677662

[pbi12780-bib-0008] Farid, S. (2007) Process economics of industrial monoclonal antibody manufacture. J. Chromatogr. B Analyt. Technol. Biomed. Life Sci. 848, 8–18. 10.1016/j.jchromb.2006.07.037 16899415

[pbi12780-bib-0009] Fischer, R. , Schillberg, S. , Hellwig, S. , Twyman, R.M. and Drossard, J. (2012) GMP issues for recombinant plant‐derived pharmaceutical proteins. Biotechnol. Adv. 30, 434–439. 21856403 10.1016/j.biotechadv.2011.08.007

[pbi12780-bib-0010] Fischer, R. , Schillberg, S. , Buyel, J.F. and Twyman, R.M. (2013) Commercial aspects of pharmaceutical protein production in plants. Curr. Pharm. Des. 19, 5471–5477. 23394566 10.2174/1381612811319310002

[pbi12780-bib-0011] Gutiérrez, S.P. , Saberianfar, R. , Kohalmi, S.E. and Menassa, R. (2013) Protein body formation in stable transgenic tobacco expressing elastin‐like polypeptide and hydrophobin fusion proteins. BMC Biotechnol. 13, 40. 23663656 10.1186/1472-6750-13-40 PMC3659085

[pbi12780-bib-0012] Hakanpää, J. , Linder, M. , Popov, A. , Schmidt, A. and Rouvinen, J. (2006a) Hydrophobin HFBII in detail: ultrahigh‐resolution structure at 0.75Å. Acta Crystallogr. D Biol. Crystallogr. 62, 356–367. 16552136 10.1107/S0907444906000862

[pbi12780-bib-0013] Hakanpää, J. , Szilvay, G.R. , Kaljunen, H. , Maksimainen, M. , Linder, M. and Rouvinen, J. (2006b) Two crystal structures of Trichoderma reesei hydrophobin HFBI—The structure of a protein amphiphile with and without detergent interaction. Protein Sci. 15, 2129–2140. 16882996 10.1110/ps.062326706 PMC2242604

[pbi12780-bib-0014] Harris, L.J. and Gleddie, S.C. (2001) A modified Rp13 gene from rice confers tolerance of the Fusarium graminearum mycotoxin deoxynivalenol to transgenic tobacco. Physiol. Mol. Plant Pathol. 58, 173–181.

[pbi12780-bib-0015] Hellwig, S. , Drossard, J. , Twyman, R.M. and Fischer, R. (2004) Plant cell cultures for the production of recombinant proteins. Nat. Biotechnol. 22, 1415–1422. 15529167 10.1038/nbt1027

[pbi12780-bib-0016] Holland, T. , Sack, M. , Rademacher, T. , Schmale, K. , Altmann, F. , Stadlmann, J. , Fischer, R. *et al*. (2010) Optimal nitrogen supply as a key to increased and sustained production of a monoclonal full‐size antibody in BY‐2 suspension culture. Biotechnol. Bioeng. 107, 278–289. 20506104 10.1002/bit.22800

[pbi12780-bib-0017] Hood, E.E. , Gelvin, S.B. , Melchers, L.S. and Hoekema, A. (1993) New Agrobacterium helper plasmids for gene transfer to plants. Transgenic Res. 2, 208–218.

[pbi12780-bib-0018] Höök, F. , Kasemo, B. , Nylander, T. , Fant, C. , Sott, K. and Elwing, H. (2001) Variations in coupled water, viscoelastic properties, and film thickness of a Mefp‐1 protein film during adsorption and cross‐linking: A quartz crystal microbalance with dissipation monitoring, ellipsometry, and surface plasmon resonance study. Anal. Chem. 73, 5796–5804. 11791547 10.1021/ac0106501

[pbi12780-bib-0019] Jacquet, N. , Navarre, C. , Desmecht, D. and Boutry, M. (2014) Hydrophobin fusion of an influenza virus hemagglutinin allows high transient expression in Nicotiana benthamiana, easy purification and immune response with neutralizing activity. PLoS ONE, 9, e115944. 25541987 10.1371/journal.pone.0115944 PMC4277400

[pbi12780-bib-0020] Joensuu, J.J. , Conley, A.J. , Lienemann, M. , Brandle, J.E. , Linder, M.B. and Menassa, R. (2010) Hydrophobin Fusions for High‐Level Transient Protein Expression and Purification in Nicotiana benthamiana. Plant Physiol. 152, 622–633. 20018596 10.1104/pp.109.149021 PMC2815860

[pbi12780-bib-0021] Kay, R. , Chan, A. , Daly, M. and McPherson, J. (1987) Duplication of CaMV 35S promoter sequences creates a strong enhancer for plant genes. Science 236, 1299–1302. 17770331 10.1126/science.236.4806.1299

[pbi12780-bib-0022] Kirchhoff, J. , Raven, N. , Boes, A. , Roberts, J.L. , Russell, S. , Treffenfeldt, W. , Fischer, R. *et al*. (2012) Monoclonal tobacco cell lines with enhanced recombinant protein yields can be generated from heterogeneous cell suspension cultures by flow sorting. Plant Biotechnol. J. 10, 936–944. 22758383 10.1111/j.1467-7652.2012.00722.x

[pbi12780-bib-0023] Kurppa, K. , Hytönen, V.P. , Nakari‐Setälä, T. , Kulomaa, M.S. and Linder, M.B. (2014) Molecular engineering of avidin and hydrophobin for functional self‐assembling interfaces. Colloids Surf. B. Biointerfaces 120, 102–109. 24905684 10.1016/j.colsurfb.2014.05.010

[pbi12780-bib-0024] Lahtinen, T. , Linder, M.B. , Nakari‐Setälä, T. and Oker‐Blom, C. (2008) Hydrophobin (HFBI): a potential fusion partner for one‐step purification of recombinant proteins from insect cells. Protein Expr. Purif. 59, 18–24. 18267368 10.1016/j.pep.2007.12.014

[pbi12780-bib-0025] Linder, M.B. (2009) Hydrophobins: proteins that self assemble at interfaces. Curr. Opin. Colloid Interface Sci. 14, 356–363.

[pbi12780-bib-0026] Linder, M. , Selber, K. , Nakari‐Setälä, T. , Qiao, M. , Kula, M.‐R. and Penttilä, M. (2001) The hydrophobins HFBI and HFBII from Trichoderma reesei showing efficient interactions with nonionic surfactants in aqueous two‐phase systems. Biomacromol 2, 511–517. 10.1021/bm0001493 11749214

[pbi12780-bib-0027] Linder, M. , Szilvay, G.R. , Nakari‐Setälä, T. , Söderlund, H. and Penttilä, M. (2002) Surface adhesion of fusion proteins containing the hydrophobins HFBI and HFBII from Trichoderma reesei. Protein Sci. 9, 2257–2266. 10.1110/ps.0207902 PMC2373586 12192081

[pbi12780-bib-0028] Linder, M.B. , Qiao, M. , Laumen, F. , Selber, K. , Hyytiä, T. , Nakari‐Setälä, T. and Penttilä, M.E. (2004) Efficient purification of recombinant proteins using hydrophobins as tags in surfactant‐based two‐phase systems. Biochemistry 43, 11873–11882. 15362873 10.1021/bi0488202

[pbi12780-bib-0029] Mandal, M.K. , Fischer, R. , Schillberg, S. and Schiermeyer, A. (2014) Inhibition of protease activity by antisense RNA improves recombinant protein production in Nicotiana tabacum cv. Bright Yellow 2 (BY‐2) suspension cells. Biotechnol. J. 9, 1065–1073. 24828029 10.1002/biot.201300424

[pbi12780-bib-0030] Mason, H.S. , Guerrero, F.D. , Boyer, J.S. and Mullet, J.E. (1988) Proteins homologous to leaf glycoproteins are abundant in stems of dark‐grown soybean seedlings. Analysis of proteins and cDNAs. Plant Mol. Biol. 11, 845–856. 24272634 10.1007/BF00019524

[pbi12780-bib-0031] McLean, M.D. , Chen, R.J. , Yu, D.Q. , Mah, K.Z. , Teat, J. , Wang, H.F. , Zaplachinski, S. *et al*. (2012) Purification of the therapeutic antibody trastuzumab from genetically modified plants using safflower Protein A‐oleosin oilbody technology. Transgenic Res. 21, 1291–1301. 22382463 10.1007/s11248-012-9603-5

[pbi12780-bib-0032] Mustalahti, E. , Saloheimo, M. and Joensuu, J.J. (2013) Intracellular protein production in Trichoderma reesei (Hypocrea jecorina) with hydrophobin fusion technology. New Biotechnol. 30, 262–268. 10.1016/j.nbt.2011.09.006 21971507

[pbi12780-bib-0033] Nagata, T. and Kumagai, F. (1999) Plant cell biology through the window of the highly synchronized tobacco BY‐2 cell line. Methods Cell Sci. 21, 123–127. 10728644 10.1023/a:1009832822096

[pbi12780-bib-0035] Pereira, E. , Kolotilin, I. , Conley, A. and Menassa, R. (2014) Production and characterization of in planta transiently produced polygalacturonase from Aspergillus niger and its fusions with hydrophobin or ELP tags. BMC Biotechnol. 14, 59. 24970673 10.1186/1472-6750-14-59 PMC4083859

[pbi12780-bib-0036] Phan, H.T. , Hause, B. , Hause, G. , Arcalis, E. , Stoger, E. , Maresch, D. , Altmann, F. *et al*. (2014) Influence of elastin‐like polypeptide and hydrophobin on recombinant hemagglutinin accumulations in transgenic tobacco plants. PLoS ONE 9, e99347. 24914995 10.1371/journal.pone.0099347 PMC4051685

[pbi12780-bib-0037] Raven, N. , Rasche, S. , Kuehn, C. , Anderlei, T. , Klöckner, W. , Schuster, F. , Henquet, M. *et al*. (2015) Scaled‐up manufacturing of recombinant antibodies produced by plant cells in a 200‐L orbitally‐shaken disposable bioreactor. Biotechnol. Bioeng. 112, 308–321. 25117428 10.1002/bit.25352

[pbi12780-bib-0038] Reuter, L.J. , Bailey, M.J. , Joensuu, J.J. and Ritala, A. (2014) Scale‐up of hydrophobin‐assisted recombinant protein production in tobacco BY‐2 suspension cells. Plant Biotechnol. J. 12, 402–410. 24341724 10.1111/pbi.12147

[pbi12780-bib-0039] Ritala, A. , Häkkinen, S. and Schillberg, S. (2014) Molecular pharming in plants and plant cell cultures: a great future ahead? Pharm. Bioprocess. 2, 223–226.

[pbi12780-bib-0040] Saberianfar, R. , Joensuu, J.J. , Conley, A.J. and Menassa, R. (2015) Protein body formation in leaves of Nicotiana benthamiana: a concentration‐dependent mechanism influenced by the presence of fusion tags. Plant Biotechnol. J. 13, 927–937. 25640969 10.1111/pbi.12329

[pbi12780-bib-0041] Sarparanta, M. , Bimbo, L.M. , Rytkoänen, J. , Mäkilä, E. , Laaksonen, T.J. , Laaksonen, P. , Nyman, M. *et al*. (2012) Intravenous delivery of hydrophobin‐functionalized porous silicon nanoparticles: stability, plasma protein adsorption and biodistribution. Mol. Pharm. 9, 654–663. 22277076 10.1021/mp200611d

[pbi12780-bib-0042] Silhavy, D. , Molnar, A. , Lucioli, A. , Szittya, G. , Hornyik, C. , Tavazza, M. and Burgyan, J. (2002) A viral protein suppresses RNA silencing and binds silencing‐generated, 21‐ to 25‐nucleotide double‐stranded RNAs. EMBO J. 21, 3070–3080. 12065420 10.1093/emboj/cdf312 PMC125389

[pbi12780-bib-0500] Soikkeli, M. , Kurppa, K. , Kainlauri, M. , Arpiainen, S. , Paananen, A. , Gunnarsson, D. , Joensuu, J.J. , *et al*. (2016) Graphene Biosensor Programming with Genetically Engineered Fusion Protein Monolayers ACS Appl. Mat. Interf. 8, 8257–8264. 10.1021/acsami.6b00123 26960769

[pbi12780-bib-0043] Sunde, M. , Kwan, A.H.Y. , Templeton, M.D. , Beever, R.E. and Mackay, J.P. (2008) Structural analysis of hydrophobins. Micron. 39, 773–784. 17875392 10.1016/j.micron.2007.08.003

[pbi12780-bib-0044] Szilvay, G.R. , Paananen, A. , Laurikainen, K. , Vuorimaa, E. , Lemmetyinen, H. , Peltonen, J. and Linder, M.B. (2007) Self‐assembled hydrophobin protein films at the air‐water interface: structural analysis and molecular engineering. Biochemistry 46, 2345–2354. 17297923 10.1021/bi602358h

[pbi12780-bib-0045] Uhlen, M. , Guss, B. , Nilsson, B. , Gatenbeck, S. , Philipson, L. and Lindberg, M. (1984) Complete sequence of the staphylococcal gene encoding protein A. A gene evolved through multiple duplications. J. Biol. Chem. 259, 1695–1702. 6319407

[pbi12780-bib-0046] Wessels, J.G.H. (1994) Developmental Regulation of Fungal Cell‐Wall Formation. Ann. Rev. Phytopathol. 32, 413–437.

[pbi12780-bib-0047] Wosten, H.A.B. and Scholtmeijer, K. (2015) Applications of hydrophobins: current state and perspectives. Appl. Microbiol. Biotechnol. 99, 1587–1597. 25564034 10.1007/s00253-014-6319-x

[pbi12780-bib-0048] Wu, K. , Malik, K. , Tian, L. , Hu, M. , Martin, T. , Foster, E. , Brown, D. *et al*. (2001) Enhancers and core promoter elements are essential for the activity of a cryptic gene activation sequence from tobacco, tCUP. Mol. Genet. Genomics 265, 763–770. 11523793 10.1007/s004380100478

[pbi12780-bib-0049] Yang, L. , Biswas, M.E. and Chen, P. (2003) Study of binding between protein A and immunoglobulin G using a surface tension probe. Biphys. J. 84, 509–522. 10.1016/S0006-3495(03)74870-X PMC1302631 12524303

