# Supplementary material for: In‐solution antibody harvesting with a plant‐produced hydrophobin–Protein A fusion
Source: Plant Biotechnol J. 2017 Aug 1;16(2):404–14. doi: 10.1111/pbi.12780 (PMC5787837; doi:10.1111/pbi.12780)
Supplement: Supplementary file 1 — Figure S1 Nucleotide sequences of expression cassettes. [file PBI-16-404-s002.pdf]

a)

Prb1 SS … *C-myc*

ATGGGATTTTTTCTCTTTTCACAAATGCCCTCATTTTTTCTTGTCTCTACACTTCTCTTATTCCTAATAATATCTCACTCTTCTCATGCCTCTAGAGAGC

HFB1

AGAAGTTGATTTCTGAGGAGGATCTTAGCAACGGCAACGGCAATGTTTGCCCTCCCGGCCTCTTCAGCAACCCCCAGTGCTGTGCCACCCAAGTCCTTGG

CCTCATCGGCCTTGACTGCAAAGTCCCCTCCCAGAACGTTTACGACGGCACCGACTTCCGCAACGTCTGCGCCAAAACCGGCGCTCAGCCTCTCTGCTGC

linker

GTGGCCCCCGTTGCCGGCCAGGCTCTTCTGTGCCAGACCGCCGTCGGTGCTGGTGCTGGTGGAGGCTCTGGTGGAGGCTCAGGTGGAGGCAGTGCAGGAA

ProteinA

ATGCTGCACAGCATGATGAAGCTCAACAGAACGCATTCTACCAAGTTCTTAACATGCCTAATTTGAACGCTGATCAGAGAAATGGTTTCATTCAATCTCT

N→Q

TAAGGATGATCCATCTCAGTCAGCTAACGTGTTAGGAGAAGCACAAAAGCTC**CAG**GATTCACAAGCTCCAAAAGCTGATGCACAACAGAATAAGTTTAAC

AAAGATCAACAGAGTGCATTCTACGAGATCCTCAACATGCCTAATCTCAACGAAGAGCAGAGAAACGGTTTTATCCAATCTCTTAAAGATGATCCAAGTC

N→Q

AGTCTACTAATGTTCTTGGAGAAGCTAAGAAATTG**CAG**GAGTCACAAGCTCCTAAGGCAGATAATAACTTCAATAAGGAACAGCAAAACGCATTCTACGA

GATCTTGAATATGCCTAATTTGAACGAAGAGCAGAGGAACGGTTTCATCCAAAGTCTTAAAGATGATCCTTCACAAAGTGCTAATCTTTTGGCTGAAGCA

N→Q

AAGAAATTG**CAG**GAGTCTCAAGCTCCAAAGGCAGATAATAAGTTTAACAAAGAACAACAGAACGCTTTCTACGAGATTCTTCATTTGCCTAATCTTAACG

AAGAGCAGAGAAATGGTTTTATTCAATCACTTAAAGATGATCCTTCTCAGTCAGCTAATCTTCTCGCTGAGGCAAAGAAATTG**AAC**GATGCTCAAGCACC

N→Q

TAAGGCTGACAATAAGTTTAACAAAGAGCAACAGAATGCATTCTACGAGATTTTACACCTCCCT**CAG**TTGACAGAAGAGCAAAGGAATGGTTTTATTCAA

Strep … *KDEL*

AGTCTTAAGGATGATCCAAGTGTGTCTAAAGAAATCTTAGCAGAAGCAAAGAAATTGAATGATGCTTGGTCCCACCCTCAGTTCGAGAAGAAGGATGAGC

TT

b)

Prb1 SS … *C-myc*

ATGGGATTTTTTCTCTTTTCACAAATGCCCTCATTTTTTCTTGTCTCTACACTTCTCTTATTCCTAATAATATCTCACTCTTCTCATGCCTCTAGAGAGC

HFB2

AGAAGTTGATTTCTGAGGAGGATCTTGCTGTTTGCCCTACTGGATTGTTCTCTAACCCTTTATGTTGTGCTACTAATGTGTTGGATTTGATTGGTGTGGA

TTGTAAGACTCCTACTATTGCTGTTGATACAGGTGCAATCTTTCAAGCTCATTGTGCATCTAAGGGATCAAAACCTCTTTGCTGTGTTGCTCCAGTTGCA

linker … *ProteinA*

GATCAGGCTTTATTATGCCAGAAGGCTATTGGTACTTTCGGTGCTGGTGGAGGCTCTGGTGGAGGCTCAGGTGGAGGCAGTGCAGGAAATGCTGCACAGC

ATGATGAAGCTCAACAGAACGCATTCTACCAAGTTCTTAACATGCCTAATTTGAACGCTGATCAGAGAAATGGTTTCATTCAATCTCTTAAGGATGATCC

N→Q

ATCTCAGTCAGCTAACGTGTTAGGAGAAGCACAAAAGCTC**CAG**GATTCACAAGCTCCAAAAGCTGATGCACAACAGAATAAGTTTAACAAAGATCAACAG

AGTGCATTCTACGAGATCCTCAACATGCCTAATCTCAACGAAGAGCAGAGAAACGGTTTTATCCAATCTCTTAAAGATGATCCAAGTCAGTCTACTAATG

N→Q

TTCTTGGAGAAGCTAAGAAATTG**CAG**GAGTCACAAGCTCCTAAGGCAGATAATAACTTCAATAAGGAACAGCAAAACGCATTCTACGAGATCTTGAATAT

N→Q

GCCTAATTTGAACGAAGAGCAGAGGAACGGTTTCATCCAAAGTCTTAAAGATGATCCTTCACAAAGTGCTAATCTTTTGGCTGAAGCAAAGAAATTG**CAG**

GAGTCTCAAGCTCCAAAGGCAGATAATAAGTTTAACAAAGAACAACAGAACGCTTTCTACGAGATTCTTCATTTGCCTAATCTTAACGAAGAGCAGAGAA

ATGGTTTTATTCAATCACTTAAAGATGATCCTTCTCAGTCAGCTAATCTTCTCGCTGAGGCAAAGAAATTG**AAC**GATGCTCAAGCACCTAAGGCTGACAA

N→Q

TAAGTTTAACAAAGAGCAACAGAATGCATTCTACGAGATTTTACACCTCCCT**CAG**TTGACAGAAGAGCAAAGGAATGGTTTTATTCAAAGTCTTAAGGAT

Strep … *KDEL*

GATCCAAGTGTGTCTAAAGAAATCTTAGCAGAAGCAAAGAAATTGAATGATGCTTGGTCCCACCCTCAGTTCGAGAAGAAGGATGAGCTT

Figure S1. Nucleotide sequences of expression cassettes for a) HFBI-ProteinA and b) HFBII-ProteinA. Genes for HFB, linker and ProteinA were cloned in the vector between BsaI restriction sites using Golden gate assembly. The gene of interest is placed under control of double the 35S promoter and the vsp terminator. A Pr1b signal sequence (MGFFLFSQMPSFFLVSTLLLFLIISHSSHASR) directs the protein to secretory pathway and a KDEL-signal retains it in the ER. The vector also introduces a codon optimized C-myc-tag (GAGCAGAAGTTGATTTCTGAGGAGGATCTT) in the N-terminus and a codon optimized StrepII-tag (TGGTCCCACCCTCAGTTCGAGAAG) in the C-terminus of the amino acid sequence.
