# Supplementary material for: In‐solution antibody harvesting with a plant‐produced hydrophobin–Protein A fusion
Source: Plant Biotechnol J. 2017 Aug 1;16(2):404–14. doi: 10.1111/pbi.12780 (PMC5787837; doi:10.1111/pbi.12780)
Supplement: Supplementary file 2 — Figure S2 QCM‐D experiment showing antibody binding to HFBI‐Protein A layer. [file PBI-16-404-s001.pdf]

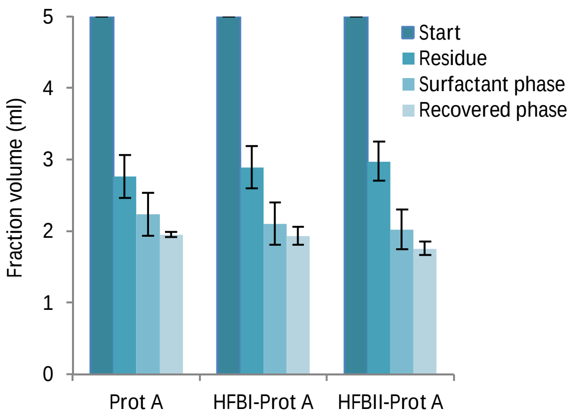

Figure S2. Volumes of fraction recovered from ATPS in Figure 3. Error bars indicate standard deviation (n=3).
